# The Impact of Thrombophilic Factors on Disease Progression in Children with Biliary Atresia—A Single-Centre Cohort Study

**DOI:** 10.3390/jcm12062108

**Published:** 2023-03-08

**Authors:** Johanna Ohlendorf, Hella Kiene, Jessica Wiegandt, André Karch, Veronika K. Jaeger, Tobias Laue, Norman Junge, Frauke Mutschler, Imeke Goldschmidt, Eva-Doreen Pfister, Christoph Leiskau, Claus Petersen, Omid Madadi-Sanjani, Joachim Friedrich Kuebler, Juliane Katharina Götz, Ulrich Baumann

**Affiliations:** 1 Paediatric Gastroenterology, Hepatology and Liver Transplantation, Hanover Medical School, 30625 Hanover, Germany; 2 Institute of Epidemiology and Social Medicine, University of Muenster, 48149 Muenster, Germany; 3 Department of Paediatrics and Adolescent Medicine, University Medical Centre Goettingen, Georg August University, 37075 Goettingen, Germany; 4 Paediatric Surgery, Hannover Medical School, 30625 Hanover, Germany; 5 Institute of Immunology and Immunotherapy, University of Birmingham, Birmingham B15 2TT, UK

**Keywords:** biliary atresia, child, male, liver cirrhosis, paediatric liver transplantation, risk factors, thrombophilia, MTHFRnt677TT

## Abstract

Epidemiological evidence suggests that thrombophilic factors, including male sex, non-O blood type, MTHFRnt677TT mutation, factor V Leiden G1691A mutation, and prothrombin G20210A polymorphism, may contribute to the progression of fibrosis and occurrence of portal vein thrombosis in liver disease. We retrospectively investigated the effect of potentially thrombophilic factors on native liver survival as a patient-relevant endpoint of disease progression in a cohort of 142 children being followed up for biliary atresia at Hannover Medical School from April 2017 to October 2019. No significant association could be determined. There was no evidence for relevant differences in native liver survival for the Factor V Leiden G1691A mutation (hazard ratio [HR] = 0.86, 95% confidence interval [CI] 0.38–1.98, *p* = 0.73), prothrombin G20210A polymorphism (HR = 0.96, 95%CI 0.24–3.65, *p* = 0.96), non-O blood type (HR = 0.79, 95%CI 0.51–1.21, *p* = 0.28) or MTHFRnt677TT mutation (HR = 1.24, 95%CI 0.60–2.56, *p* = 0.56). A certain, albeit not strong, evidence of reduced native liver survival in male patients after Kasai hepatoportoenterostomy, particularly during the first 2000 days (42%; HR = 1.41, 95%CI 0.92–2.18, *p* = 0.11) was found. All children with pre-transplant portal vein thrombosis (*n* = 7) had non-O blood types. Larger multi-centre studies are necessary to show if the male sex or other thrombophilic factors could be potentially associated with reduced native liver survival.

## 1. Introduction

Biliary atresia (BA) is the most frequent indication for paediatric liver transplantation (LT) [1]. Kasai hepatoportoenterostomy (HPE) may reinstate bile flow and prevent or delay LT [2]. Only 18–51% of patients with BA live with their native liver by the age of 20 years [2,3,4]. Various factors have been associated with the progression of liver fibrosis and the shortening of native liver survival (NLS) [2,5,6,7,8].

The coagulation system is involved in fibrogenesis in patients with liver disease [9]. The Factor V Leiden G1691A mutation (FVL) [10], prothrombin G20210A polymorphism (FIIG20210A) [11], non-O blood types (non-O BT) of the ABO blood type system [12], MTHFRnt677TT mutation [13], and male sex [14] have been suggested to accelerate the progression of liver fibrosis [9,15]. In adult patients with a chronic HCV infection, an association of the presence of FVL or FIIG20210A with more rapid liver fibrosis progression was reported [16,17,18]. More severe liver fibrosis has been observed in patients with non-O BT [19,20]. The influence of all the above-mentioned factors on the development of clinically relevant liver fibrosis in the general population has also been shown [9]. A link between the presence of cryptogenic or alcoholic liver cirrhosis and the occurrence of the MTHFR677TT mutation was detected [21]. Furthermore, patient sex has an effect on the progression of liver fibrosis [22,23,24]. An association between thrombophilic genetic factors and the development of portal vein thrombosis (PVT) has been described [21,25,26].

This study aimed to investigate the effect of potentially thrombophilic risk factors on reduced NLS and the occurrence of pre-transplant PVT in children with BA.

## 2. Materials and Methods

### 2.1. Study Design and Participants

**Figure 1:**
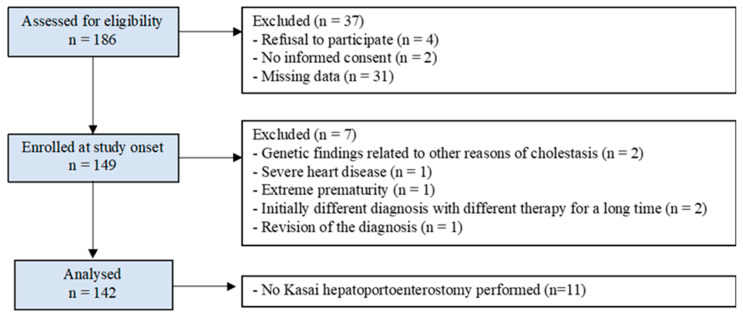
Patient flow diagram.

This was a single-centre retrospective cohort study. Children with BA who were seen during planned clinical visits in our hospital from April 2017 to November 2019 were included and analysed in this study (Figure 1). All children were followed up for at least 2 years. The inclusion criteria were a diagnosis of BA and informed consent from parents and/or caregivers. Diagnoses were confirmed by specific results of hepatobiliary sequence scintigraphy and/or endoscopic retrograde cholangiopancreatography, characteristic intraoperative cholangiography, distinctive pretransplant liver biopsies, or typical liver explants.

The exclusion criteria were missing data on prothrombotic factors, loss to follow-up, comorbidities with other diseases with decisive effects on the progression of liver fibrosis (genetic findings related to other reasons for cholestasis and severe heart disease), extreme prematurity, different initial diagnosis with other therapies, and the absence of informed consent. Children whose diagnoses of BA had to be revised were retrospectively excluded.

Thrombophilic risk factors were assessed as part of routine laboratory surveillance and included genotyping for FVL, FIIG20210A, and MTHFRnt677TT mutation.

The Ishak liver fibrosis score (IFS) was determined using the histology reports of all biopsies performed at the time of HPE/diagnosis and from the liver explants after LT. The occurrence of PVT and portal vein hypoplasia (portal vein diameter ≤ 3 mm) was identified using sonography findings and the LT operation reports. The anatomical BA type, classified by the schematic of Ohi type [8], and the presence of cystic BA [27] were determined using the HPE operation reports.

Further patient characteristics included sex, BT, prematurity, presence of associated extrahepatic anomalies (e.g., BA splenic malformations (BASM), cardiac-associated BA (CABA), and heterotaxy syndrome), cytomegalovirus (CMV) infection (CMV-IgM detection at the time of HPE), and age at HPE and at LT. Laboratory values included total serum bilirubin (TSB) concentration from 4 weeks to 3 months after HPE, TSB concentration 3–6 months after HPE, and normalisation of TSB after HPE while NLS. Moreover, complications after HPE (complicated clinical courses, such as relaparotomy, anastomosis or suture insufficiency, bleeding complications, and bride ileus), the presence of postoperative cholangitis after HPE or recurrent cholangitis and the medication were evaluated [28]. All baseline characteristics were retrospectively obtained from patient notes.

The study was approved by the Ethics Committee of Hanover Medical School (Statement No. 3492) and performed in accordance with the Helsinki Declaration on medical research involving human subjects. Written informed consent was obtained from parents and/or caregivers.

### 2.2. Endpoints

The primary endpoint was NLS, which began at birth and ended at either death or LT. Because of the critical effect of HPE on NLS, patients with primary LT were excluded from this analysis. To assess the progression of liver fibrosis, the IFS at the time of HPE/diagnosis were analysed as the secondary endpoint. The third endpoint was the normalisation of TSB after HPE and before LT. Patients who underwent primary LT were excluded from this part of the analysis. Finally, the development of pre-transplant PVT was analysed as the fourth endpoint.

The occurrence of the male sex, FVL, FIIG20210A, MTHFRnt677TT mutation, the non-O BT, age at HPE, prematurity, anatomical BA type III, cystic BA, BASM, CABA, presence of heterotaxy syndrome, CMV association (CMV IgM+), complicated clinical course, postoperative cholangitis, recurrent cholangitis, and use of medication were used as exposures.

### 2.3. Statistical Analysis

Baseline characteristics are described as absolute numbers and proportions for nominal variables, medians with interquartile ranges for ordinal variables, and medians with interquartile ranges and means with standard deviations for continuous variables. NLS was displayed using Kaplan–Meier plots and compared between groups using univariable and multivariable Cox regression models (adjusted for sex). Early progression of liver fibrosis as measured by the IFS at HPE/diagnosis and the normalisation of serum bilirubin concentration after HPE and before LT were analysed using an (ordered) logistic regression model. Missing data were not imputed. If data were missing, the patients were excluded from the respective partial analysis. Statistical analyses were performed using Stata, version 14 and Microsoft Excel, version 16.37.

## 3. Results

### 3.1. Characteristics of Study Population

A total of 142 children (82 girls and 60 boys) aged 2–23 years (median 11.5 years) with BA were included and analysed. The baseline characteristics of the participants are summarised in Table 1.

The patients’ ages at the time of HPE ranged from 16 to 113 days (median, 56 days). Eleven patients (8%) did not undergo an HPE because of late diagnosis and/or cirrhosis found intraoperatively (*n* = 8), cirrhosis on liver biopsy (*n* = 2), or unknown reasons (*n* = 1).

Sixty-six (51%) patients achieved TSB normalisation after HPE. Sixty-eight children (53%) had an IFS of ≥4 at the time of HPE/diagnosis, suggesting rapid progression of liver fibrosis.

Seven children (5%) developed PVT before LT. As a long-term medication, 96% of the patients received ursodeoxycholic acid and fat-soluble vitamins. Antibiotic therapy after HPE/open cholangiography and subsequent prophylaxis for at least 6 months was administered in 93% of the children. Fifty-four (42%) patients received postoperative steroid therapy after HPE.

Seventy-three per cent of the children with BA had undergone LT. The ages at LT ranged from 133 days to 10 years (median, 304 days). At the time of LT, 99% of the patients had advanced fibrosis or cirrhosis (IFS 5 or 6).

**Figure 2:**
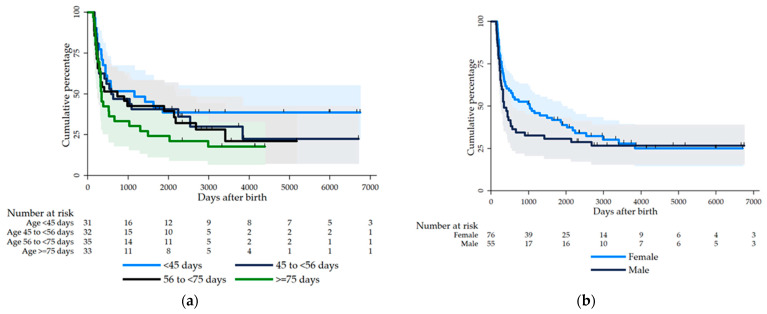
(**a**) Association of timing of HPE with NLS. Kaplan–Meier curves with time from birth until LT in children with BA, stratified by age at HPE, divided into four groups. (**b**) Association of sex with NLS. Kaplan–Meier curves from birth until LT in children with BA, stratified by sex (HR = 1. 41, CI 95% 0.91–2.18; *p* = 0.11)**.**

Patients with a shorter NLS more frequently had a late HPE (>75 days) (Table 1, Figure 2). Children with cystic BA showed a longer NLS (Table 1). There was no significant association between the use of steroids and the anatomical type III with native liver survival.

Children with BASM, CABA and CMV association showed similar NLS and early progression of liver fibrosis as those outside these groups (Table 1). Heterotaxy syndrome, recurrent cholangitis, a complicated clinical course, or prematurity also had no relevant effect on NLS or early progression of liver fibrosis.

### 3.2. Association of Thrombophilic Factors in HPE Group (n = 131) with NLS, Liver Fibrosis at the Time of HPE/Diagnosis, and Normalisation of TSB after HPE

The proportion of the male sex was 42% (*n* = 60). The MTHFRnt677TT mutation was present in 11 patients (8%); FVL mutation, 8 patients (6%); and heterozygous FIIG20210A, 3 patients (2%). No homozygotes for FVL or the FIIG20210A were observed. Non-O BT was present in 87 patients with HPE (67%) (Table 2).

#### 3.2.1. Sex

There was a certain, albeit not strong, correlation between the male sex and earlier liver transplantation, especially during the first 2000 days (HR = 1.41, 95%CI 0.92–2.18, *p* = 0.11) (Figure 2). This was not significant but consistent in the multivariable analysis (adjusted for the timing of HPE, anatomic BA type, cystic BA, and medication with steroids (HR = 1.40, 95%CI 0.87–2.28, *p* = 0.17)). Only 42% of the boys and 57% of the girls achieved TSB normalisation after HPE. There was some non-significant evidence found that the male sex was a risk factor for persistent cholestasis (OR = 1.81, 95%CI 0.89–3.69, *p* = 0.10).

Boys showed higher TSB concentrations at the time (1) 4 weeks to 3 months after HPE and (2) 3–6 months after HPE (Table 3). This should be emphasized against the background of boys showing a lower IFS in biopsy at the time of HPE/diagnosis (OR = 0.53, 95%CI 0.28–1.00; *p* = 0.05). There was no difference in the timing of the HPE between sexes (HR = 0.92, 95%CI 0.65–1.29, *p* = 0.62).

#### 3.2.2. Non-O Blood Types

There was no significant effect of the non-O BT on the early progression of fibrosis as measured by the IFS at the time of sex-adjusted HPE (OR = 1.73, 95%CI 0.87–3.46, *p* = 0.12), particularly considering the timing of the biopsy and presence of cystic BA (OR = 1.42, 95%CI 0.69–2.90, *p* = 0.34). Non-O BT was not associated with earlier NLS (HR = 0.79, 95%CI 0.51–1.21, *p* = 0.28) or persistent cholestasis after HPE (OR = 0.91, 95%CI 0.43–1.91, *p* = 0.80).

#### 3.2.3. MTHFRnt677TT Mutation

No evidence was found in the HPE group (*n* = 131) that children with the MTHFRnt677TT mutation received LT earlier than MTHFRnt677T negative or heterozygous children in the analysis only adjusted for sex (hazard ratio [HR] = 1.24, 95%CI 0.60–2.56, *p* = 0.56). This effect, however, became stronger in multivariable analysis adjusted for sex, timing of HPE, anatomic BA type, cystic BA, and medication with steroids (HR = 2.00, 95%CI 0.90–4.45, *p* = 0.09).

There was no evidence for a relevant effect of the MTHFRnt677TT mutation on persistent cholestasis after HPE (OR = 1.33, 95%CI 0.38–4.60 *p* = 0.65) (Table 4). In addition, there was no evidence for a significant difference in the early progression of liver fibrosis as measured by the IFS at the time of HPE when the MTHFRnt677TT mutation was carried (OR = 1.21, 95%CI 0.39–3.69, *p* = 0.73).

#### 3.2.4. Factor V Leiden G1691A Mutation and Prothrombin G20210A Polymorphism

There was no evidence for relevant differences in NLS for FVL (HR = 0.86, 95%CI 0.38–1.98, *p* = 0.73) or FIIG20210A (HR = 0.96, 95%CI 0.24–3.65, *p* = 0.96). The same applies to the multivariable analysis.

Patients with FVL mutations showed rather a lower degree of fibrosis at the time of HPE (OR = 0.35, 95%CI 0.09–1.39, *p* = 0.11) without significant difference in TSB levels after HPE (OR = 1.0, 95%CI 0.26–4.55 *p* = 0.91). Two of the children with the FIIG20210A already had an IFS of ≥5.5 at the time of HPE, and the third had an IFS of 2.5.

### 3.3. Thrombophilic Factors in the Children with PVT

The seven children with pre-transplant PVT had non-O BT; six of them received HPE; one carried the MTHFRnt677TT mutation. Only two of these patients had portal vein hypoplasia (Appendix A Table A1). There was no evidence of pre-transplant portal vein thrombosis and earlier LT (HR 1.46, 95%CI 0.67–1.92, *p* = 0.37). None of the PVT patients received special treatment for thrombosis. Liver function at the time of thrombosis was very heterogeneous in the patients, ranging from normal to impaired. In three patients, PVT was discovered intraoperatively during LT. PVT was diagnosed by computed tomography in one patient and by ultrasonography in the other three patients. They all received liver transplantation within 4–8 weeks of diagnosis of PVT.

## 4. Discussion

Previous studies have suggested that the presence of thrombophilic factors is associated with an increased rate of liver fibrosis progression and more frequent PVT in adults with chronic liver diseases [9,16,17,18,19,20,21,24,25,26]. In this single-centre homogenous cohort study, there was no significant association between the progression of liver fibrosis, native liver survival or persistence of cholestasis after HPE and the examined thrombophilic factors. Certain, albeit not strong, evidence of reduced native liver survival in male patients after Kasai hepatoportoenterostomy, particularly during the first 2000 days, was found. All children with pre-transplant portal vein thrombosis (*n* = 7) had non-O blood types. In line with the findings of previous studies, children with earlier HPE and cystic BA lived longer with their native livers [2,27]. In summary, larger multi-centre studies are necessary to show if the male sex or other thrombophilic factors could be potentially associated with reduced native liver survival.

In our study, there was certain, albeit not strong, evidence of reduced native liver survival in male patients, especially during the first 2000 days. This was not significant but consistent in the multivariable analysis. Only 42% of the boys achieved TSB normalisation after HPE; certainly, the male sex was not a risk factor for persistent cholestasis.

The effect of the male sex on the progression of liver fibrosis in adults with chronic liver disease, such as chronic HBV infection, has already been demonstrated. One possible reason for this is the protective effect of female sex hormones on inflammation-related fibrosis [22,24]. This connection is unlikely in childhood because of the lower sex hormone levels in prepubescent children. In addition, the male sex is a known risk factor for recurrent thromboembolism, suspecting genetic associations [14,29]. It is interesting that in our patients, the male sex appeared to be a protective factor in delaying early progression, while in the long term, it potentially led to an earlier LT. It remains unclear which factors contribute to the mechanism of sex disparity in the progression of liver fibrosis and the timing of LT in children with BA.

In our paediatric cohort, there was no significant effect of the non-O BT on the early progression of fibrosis, native liver survival or persistent cholestasis after HPE. The non-O BT is associated with increased von Willebrand factor (vWF) and increased factor VIII levels, which cause thrombophilia [12,30]. It is critical to note that we did not measure vWF or factor VIII levels. However, the severity of the cirrhotic liver disease is also associated with increased vWF levels [31]. The latter association could overrule the effect of the non-O BT, especially in more advanced stages of the disease [31].

Seven children in this study population developed a pre-transplant PVT. All of them had non-O BT. Previous studies found an association between thrombophilic factors and the development of PVT in adults [21,25,26]. Contrasting studies explained missing effects, with cirrhosis being an independent risk factor for PVT overruling the effects [31]. Caruso et al. found that 15% of children with BA developed a PVT, but only children close to LT were analysed [32]. Of the seven children with PVT, only two suffered from portal vein hypoplasia. The portal vein hypoplasia is to be seen as a consequence of advanced liver fibrosis, as described for 26% of the children with end-stage BA by Caruso [32,33].

A link between MTHFRnt677T mutation and the presence of cirrhosis is described in adults [21]. However, there are also controversial studies without any effects of the MTHFRnt677TT mutation on the extent or progression of liver fibrosis [18,34]. In our study, for children with BA, there was no significant association between native liver survival and the presence of the MTHFRnt677TT mutation. After taking into account known risk factors, the effect of the MTHFRnt677TT mutation became stronger while still not being significant. The potentially prothrombotic effect of the MTHFRnt677TT mutation has been described to be mediated by homocysteinaemia and influenced by folic acid levels and the supply of vitamins B6 and B12 [13,35,36]. This finding may contribute to the more recently controversial results of studies on the prothrombotic effect of the MTHFRnt677TT mutation [13,37,38].

The association between FVL and FIIG20210A with faster liver fibrosis progression in adulthood remains controversial [9,17,18,39]. For children with BA, no evidence for an effect of FVL and FIIG20210A on IFS, NLS or persistent cholestasis was found. Nevertheless, a positive correlation has been demonstrated in larger study populations, especially for the FVL [9,17]. Plompen et al. showed an effect of the non-O BT on the development of clinically relevant fibrosis only in combination with FVL or FIIG20210A [9]. Therefore, a distinct effect of non-O BT, in combination with these factors, could also be observed in a larger study population.

This was a single-centre study; therefore, the number of patients was limited. In the complex disease of biliary atresia, the influence of several progression factors and genes with small effects is likely. Nonetheless, we provided a more homogeneous patient population with respect to diagnostic processes. Due to the low power with respect to the rare occurrence of thrombophilic factors, larger multi-centre studies are necessary for a better understanding of their role in disease progression.

## 5. Conclusions

In conclusion, our data do not support a dominant role of thrombophilic factors in the disease progression in children with biliary atresia. Our data show reduced native liver survival in male patients with BA.

This is the first study to investigate the association between potentially thrombophilic factors and the progression of liver fibrosis in children with BA. Multicentre studies on the aetiology and progression factors of BA are needed for a better understanding of this complex disease [40]. This study provides a basis for future follow-up studies with larger sample sizes.

## Figures and Tables

**Table 1:** Baseline characteristics.

Characteristic		Total Cohort (*n* = 142)	NLS (HR)	95%CI	*p* Value ^$^
Male sex		60 (42%)	1.37 ^Ω^	0.92–2.07	0.12
Kasai hepatoportoenterostomy (HPE)		131 (92%)	0.31	0.17–0.59	<0.001
Age at HPE (days) *	Mean (SD)	60 ± 23.5			
	≥45–<56		1.31	0.70–2.43	0.39
	≥56–<75		1.39	0.77–2.54	0.27
	≥75		1.81	1.00–3.26	<0.05
IFS at HPE */diagnosis	Median (IQR)	4 (3–5)	1.22	1.04–1.45	0.01
	Missing	11 (8%)			
BA type * (Ohi type)	I	30 (21%)			
	II	4 (3%)			
	III	85 (60%)	1.55 *	0.99–2.62	0.09
	Missing	23 (16%)			
Cystic BA		12 (8%)	0.36 *	0.13–0.99	<0.05
	Missing	4 (2.8%)			
Extrahepatic anomalies	BASM	32 (23%)	0.92 *	0.56–1.51	0.74
	CABA	57 (40%)	0.86 *	0.56–1.32	0.49
	Heterotaxy syndrome	8 (6%)	0.83 *	0.30–2.26	0.71
	Other syndromes	2 (1%)			
Prematurity (≤36 weeks)		13 (9%)	0.77 *	0.37–1.60	0.48
	Missing	11 (8%)			
CMV IgM+		14 (10%)	1.06 *	0.51–2.20	0.87
	Missing	17 (12%)			
Cholangitis after HPE *		63 (44%)	1.23 *	0.81–1.85	0.34
	Missing	4 (3%)			
Recurrent cholangitis *		20 (14%)	1.22 *	0.71–2.09	0.47
	Missing	3 (2%)			
Complicated clinical course after HPE *		26 (18%)	1.13 *	0.66–1.91	0.64
Medication	Ursodeoxycholic acid	137 (96%)			
	- Missing	4 (3%)			
	Fat soluble vitamins	137 (96%)			
	- Missing	5 (3.5%)			
	Antibiotics	132 (93%)			
	- Missing	5 (3.5%)			
	Steroids *	54 (38%)	0.71 *	0.46–1.09	0.11
IFS at LT ^#^	Median (IQR)	6 (6–6)			
Age of children living with native liver (years) ^§^	Mean (SD)	11 ± 6			
Normalisation of TSB after HPE	<20 µmol	66 (51%)	14.11 *	8.11–25.13	<0.001
	Missing	4 (3%)			
TSB 4 weeks–3 months after HPE (µmol) ^§^	Mean (SD)	104.1 ± 100.4	5.68 *	2.71–11.91	<0.001
	<20 µmol	28 (25%)			
	Liver transplanted	3 (2%)			
	Missing	16 (11%)			
TSB 3–6 months after HPE (µmol) ^§^	Mean (SD)	105.6 ± 132.8	9.08 *	4.96–16.60	<0.001
	<20 µmol	47 (49%)			
	Liver transplanted	23 (16%)			
	Missing	11 (11%)			
Pre-transplant portal vein hypoplasia		36 (25%)	3.71 *	2.32–5.93	<0.001
	Missing	10 (7%)			

Values are presented as absolute numbers and proportions, mean ± standard deviation, or median (interquartile range). * Only for children with a Kasai operation (HPE). ^Ω^ Restricted to the first 2000 days. ^#^ Only for children undergoing liver transplantation (LT). ^§^ Only for children with HPE and without LT at this time. ^$^ *p*-value and hazard ratio (HR) for Cox regression for transplant-free survival (NLS). BA, biliary atresia; BASM, biliary atresia splenic malformations; CABA, cardiac-associated biliary atresia; CVM, cytomegalovirus; IFS, Ishak fibrosis score; TSB, total serum bilirubin; FVL, factor V Leiden; SD, standard deviation.

**Table 2:** Occurrence of thrombophilic factors in the study group.

Parameter	Total Cohort (*n* = 142)	Children with HPE (*n* = 131)	NLS (HR)	95%CI	*p*-Value ^$^
Male sex	60 (42%)	55 (42%)	1.27 *	0.84–1.92	0.25
MTHFRnt677TT homozygous	15 (11%)	11 (8%)	1.24 *	0.60–2.56	0.56
Factor V Leiden G1691A heterozygous (FVL)	9 (6%)	8 (6%)	0.86 *	0.38–1.98	0.73
Prothrombin G20210A polymorphism (FIIG20210A) heterozygous	3 (2%)	3 (2%)	0.96 *	0.24–3.95	0.96
Blood type non-O	96 (69%)	87 (67%)	0.79 *	0.51–1.21	0.28

^$^ *p*-value and Hazard Ratio for the cox regression regarding native liver survival. * Only for those children with Kasai operation. HPE, Kasai hepatoportoenterostomy; HR, Hazard Ratio; FVL, factor V Leiden; NLS, native liver survival; FIIG20210A, Prothrombin G20210A polymorphism.

**Table 3:** Association of sex and normalisation/level of TSB after HPE.

Parameter		Boys	Girls	
Total serum bilirubin (TSB) after HPE (µmol) *	Normal (<20)	23 (42%)	43 (57%)	*n* = 127 4 missing
TSB 4 weeks–3 months after HPE (µmol) ^§^	Mean ± SD Normal (<20)	127.8 ± 113.1 8 (15%)	86.4 ± 86.5 20 (26%)	*n* = 112 16 missing 3 LT at this time
TSB 3–6 months after HPE (µmol) ^§^	Mean ± SD Normal (<20)	123.8 ± 140.1 16 (29%)	95.4 ± 128.5 31 (41%)	*n* = 97 11 missing 23 LT at this time

* Only for children with Kasai operation. ^§^ Only for children with Kasai operation and without liver transplantation at this time. TSB, total serum bilirubin; HPE, Kasai hepatoportoenterostomy; LT, liver transplantation; SD, standard deviation; IQR, interquartile range.

**Table 4:** Association of homozygous MTHFRnt677TT mutation and normalization/level of TSB after HPE.

Parameter		MTHFRnt677T Homozygous *n* = 11	MTHFRnt677T Heterozygous/Negative *n* = 120	
Age at HPE (days) *	Mean ± SD	53 ± 22	60 ± 23	*n* = 131
		4 (27%)	11 (8%)	
Total serum bilirubin (TSB) after HPE (µmol) *	Normal (<20)	5 (45.5%)	61 (50.8%)	*n* = 127
				4 missing
TSB 4 weeks–3 months after HPE (µmol) ^§^	Mean ± SD	116.9 ± 86.0	102.9 ± 102.0	*n* = 112
	Normal (<20)	3 (27.3%)	25 (20.8%)	16 missing
				3 LT at this time
TSB 3–6 months after HPE (µmol) ^§^	Mean ± SD	132.7 ± 109.9	102.8 ± 135.2	*n* = 97
	Normal (<20)	3 (27.3%)	44 (36.7%)	11 missing
				23 LT at this time

* Only for children with Kasai operation. ^§^ Only for children with Kasai operation and without liver transplantation. TSB, total serum bilirubin; HPE, Kasai hepatoportoenterostomy; LT, liver transplantation.

## Data Availability

Data are available from the corresponding author on request.

## Appendix A

**Table A1:** Characteristics of patients with pre-transplant PVT.

**Characteristic**		**Total Cohort (*n* = 7)**
Male sex		4 (57%)
Age (years)	Mean ± SD	8.6 ± 5.0
Age at portal vein thrombosis (years)	Mean ± SD	2.2 ± 2.7
Non-O blood type		7 (100%)
MTHFRnt677TT homozygous		1 (14%)
Factor V Leiden G1691A heterozygous		0
Prothrombin G20210A polymorphism heterozygous		0
Pre-transplant portal vein hypoplasia		2 (29%)

SD, standard deviation; IQR, interquartile range; PVT, portal vein thrombosis.
